# Primary Spinal Tumors and Masses in Children

**DOI:** 10.22037/ijcn.v16i2.30614

**Published:** 2022-03-14

**Authors:** Abdonaser FARZAN, Pooria AHMADI, Erfan TASDIGHI, Mahmoud Reza ZINATZADEH, Elham POURBAKHTYARAN

**Affiliations:** 1 Department of Pediatric Neurosurgery, Shahid Beheshti University of Medical Sciences,Tehran, Iran; 2 Pediatric Neurology Research Center, Research Institute for Children’s Health, Shahid Beheshti University of Medical Sciences, Tehran, Iran.; 3 Depatment Emergency Medicine, Tehran University of Medical Sciences, Tehran, Iran

**Keywords:** Spinal Cord Neoplasms, Neurosurgery, Pediatrics

## Abstract

**Objectives:**

Spinal cord tumors are rare in children, mostly presented with unspecific symptoms that might pose a problem due to their possible malignancy and further complications. However, there are limited data on spinal cord lesions in Iran. This study aimed to present a series of 37 cases of primary spinal tumors treated at the same institution and briefly review their pathology, symptoms, and site of occurrence.

**Materials & Methods:**

In this study, 37 cases of spinal cord tumors and masses were selected within March 2007-2017, excluding spinal dysraphism. The data on age, gender, clinical presentation, location of the mass, and pathology were retrospectively collected.

**Results:**

The mean age at diagnosis was 5 years and 8 months (standard deviation: 4.1 years). Moreover, 21 and 16 cases were male and female, respectively (male-to-female ratio: 1.31). Pathological findings included 9 neuroepitheliomas (i.e., 6 neuroblastoma, 2 ganglioneuroma, and 1 ganglioneuroblastoma/ganglioneuroma), 4 ependymomas, 3 primitive neuroectodermal tumors, 3 glial tumors, 4 neurodevelopmental tumors, 3 lymphomas, 1 hemangiopericytoma, and 1 neurofibroma. In addition, 26 (74.2%), 14 (40%), 6 (16.6%), and 4 (11.4%) patients had motor symptoms, pain, sensory symptoms, and urinary symptoms, respectively. The most common location of occurrence was the lumbosacral region.

**Conclusion:**

In conclusion, while differing in pathological composition and location of tumors in comparison to other papers, this study presents possible presentations and/or expected pathologies in pediatric spinal cord tumors.

## Introduction

Central nervous system (CNS) tumors are the most common solid tumors in children while being the leading cause of cancer-related death in children ≤ 14 years of age (1). The incidence of primary CNS tumors has been estimated as 3.2 (for males) and 3.9 (for females) worldwide in 100,000 individuals per year; however, it is reported to be higher in developing countries (2). Nevertheless, in children and adolescents under 19 years of age, the estimated incidence is 5.6 per 100,000 individuals per year (1). Furthermore, primary spinal tumors comprise less than 10% of all CNS tumors (3,4). 

Given the rarity of these tumors and the nonspecificity of early symptoms, diagnosis is challenging unless high suspicion is achieved (3). It is estimated that 22% of primary spinal cord tumors are malignant, and the rest are nonmalignant (5). Meningiomas, nerve sheath tumors, and ependymomas are reported to be the most common types in the general population, and astrocytomas are said to be predominant in children (6, 7). Gender discrepancy is also reported in cases of spinal masses, with provincial studies, including a systematic review, stating a male dominance; however, some western studies reported otherwise (4, 7, 8).

Still, due to their rarity, there has been a paucity of epidemiologic data on primary spinal tumors of children in Iran. The present study aimed to provide a series of cases of spinal cord neoplasms treated at the same institution and briefly review how they are presented in children with regard to their pathology, symptoms, and site of origin.

## Materials & Methods

In this study, we collected all children with a confirmed diagnosis of primary spinal cord lesion admitted to Mofid Pediatric Hospital, Tehran, Iran, in a 10-year interval within March 2007 to March 2017. The demographic data relating to the age of diagnosis and gender and medical data, including clinical presentation, location of the tumor, and pathologic classification, were retrospectively extracted from available medical records.

Secondary masses and/or recurrences were not included in the study. Moreover, duplicate cases, uncertain pathologic diagnoses, and cases with incomplete data were excluded while assessing each of the properties. For the assessment of the spinal level in which the tumor emerged, a point was given to each level that contained the mass (for masses spanning several levels of the spinal cord, a point was given to each level).

## Results

The general characteristics of patients and the main results are summarized in Table 1. Of a total of 37 patients, 21 cases were male (56.75%), and the rest 16 were female (43.25%), which led to a male-to-female ratio of 1.31. The mean age at diagnosis was 5 years and 8 months (standard deviation: 4.1 years; range: 17 days to 13 years). 

Of all presenting symptoms, motor symptoms were most frequently present in 26 children (74.28%), followed by pain which was presented in 14 patients (40%). Furthermore, 6 patients (16.6%) showed signs of sensory symptoms of either paresthesia, hypoesthesia, or anesthesia, and 4 patients (11.42%) showed urinary symptoms, which include any degree of urinary incontinence and/or retention. None of the patients was presented with scoliosis.

Of all 31 definite pathological diagnoses, the most common type was neuroepithelioma neoplasms (including neuroblastoma, ganglioneuroma, and ganglioneuroblastoma) which comprised of 9 cases (29%), followed by ependymomas (4 cases; 12.9%) and primitive neuroectodermal tumors (3 cases; 9.6%). Other encountered pathologies were neurodevelopmental tumors (i.e., dermoid cysts and sinus and epidermal inclusion cyst), hemangiopericytoma, neurofibroma, glial tumors, and lymphomas. Primary spinal cord tumors in the present study were observed all throughout the spinal cord; however, lumbosacral regions were prominently more affected.

**Figure 1:**
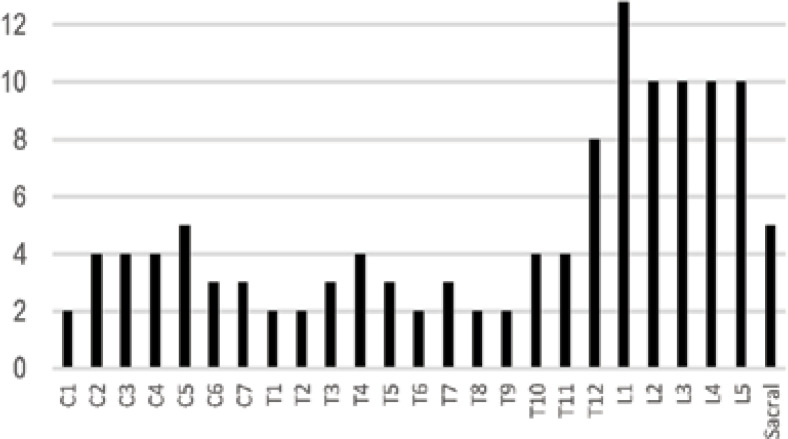
*Frequency of Spinal Cord* *Masses* *regarding Their Location*

**Table 1:** *Summary* *of* *Characteristics of 37 Cases of Spinal Cord Masses*

			Symptoms					
No.	Gender	Age (year)	Pain	Motor symptoms	Sensory symptoms	Urinary symptoms	Location	Pathology
1	F	6	+	+	-	-	T10-T12	PNET
2	F	3	-	+	-	-	T12-L1	No evidence of malignancy
3	F	2	-	+	-	+	T12-L5	
4	F	2	-	+	-	+	L1-L4	Dermoid cyst
5	M	5	+	+	-	-	T10-T12	Glioma
6	M	5	-	+	-	-	C5-C7	
7	M	8	-	-	-	+	Lumbosacral	
8	F	11	-	+	-	-	T1-T5	Ganglioneuroma
9	M	14	+	+	-	-	L2-L5	
10	F	11	+	-	-	-	L4-S1	Epidermal inclusion cyst
11	M	8 months	-	+	-	-	T12-L5	
12	M	4 months	-	-	-	-	Lumbosacral	No evidence of malignancy
13	M	9	-	+	-	-	T12-L1	
14	F	3	+	+	-	-		Neuroblastoma
15	M	11	+	-	-	-	C2-C7	Papillary ependymoma
16	M	8	-	-	-	-	L1-L5	Epidermal inclusion cyst
17	M	8	-	+	-	+		PNET
18	M	12					T1-T4	Neuroblastoma
19	F	2	-	-	-	-	L5-S1	Dermoid sinus
20	M	3	+	+	-	-		High-grade B-Cell lymphoma
21	F	11	+	+	+	-	T7-T10	Hemangioblastoma
22	F	2	-	+	-	-		Neuroblastoma
23	M	5	-	+	+	-		PNET/Medulloblastoma
24	F	4	-	+	+	-	C1-C7	Malignant round cell tumor
25	M	17 days	-	-	-	-	L5-S1	Hemangiopericytoma
26	M	8	+	-	-	-	L1-L4	Myxopapillary ependymoma
27	M	9	+	+	+	-	C1-C5	Glial tumor
28	M	6	+	+	-	-	T3-T7	Neuroblastoma
29	M	10 months	-	-	-	-	L1	Anaplastic Ependymoma
30	F	5			-		T8-T9	Ganglioneuroma
31	F	5	+	+	-	-	T11-L3	Neurofibroma
32	F	13	+	+	+	-	C2-C5, L1-L5	Schwannoma
33	M	12	+	+	-	-	T4-T7	Hodgkin’s lymphoma
34	F	4	-	+	-	-		Ganglioneuroma/Ganglioneuroblastoma
35	M	11 months	-	+	-	-		Neuroblastoma
36	F	18 months	-	+	-	-	T10-L1	Neuroblastoma
37	M	10 months	-	+	+	-		Ependymoma

F, female; M, male; PNET, primitive neuroectodermal tumors

## Discussion

Previous studies suggest a gender discrepancy in spinal tumor incidence; the present study corroborates the results of studies reporting a male dominance pattern in pediatric spinal cord tumors (4, 8, 9) but differs from other large scale studies, not confined to children, stating a female-dominant pattern (5, 7). This might be due to the age of onset, inequality of healthcare, and ethnic or geographical differences.

The patients with spinal cord masses usually have nonspecific complaints or might be asymptomatic due to the slow progression of low-grade tumors, which might considerably delay the diagnosis (9, 10). In presenting symptoms, the percentage of observed motor symptoms seems to be comparable to that of some studies; nevertheless, the current study observed a considerably lower amount of pain or sensory/urinary symptoms at diagnosis. Moreover, although scoliosis was present at diagnosis or in follow-up in several reported cases, no child with scoliosis was encountered at the time of diagnosis (8, 10).

Additionally, general population studies suggest meningioma or neurofibroma, and comparable studies mostly had astrocytoma (in one case ependymoma) as the most common type of primary spinal cord tumors (4–7, 9, 10). However, the present study encountered a prominent number of neuroblastic tumors (whether or not including ganglioneuroma and ganglioneuroblastoma). Restricting the results to intramedullary spinal tumors makes ependymoma the most common type. This might be reflected by the single-centered nature of the current study or might be due to the differences in ethnicity, age, or geographical location. 

The present study differs in a way that the location of the detected tumors was most commonly lumbosacral, whether or not including paramedullary tumors (i.e., neuroblastic tumors) but described mostly cervicothoracic in the literature (11, 12). As suggested in the literature, in our institution, total surgical resection was used as the main line of therapy where possible; however, for some cases, radiotherapy following surgery was also used (13).

## In Conclusion

Although confined by the limitations of this single-centered study, the results of a 10-year long series of a tertiary pediatric hospital shed light on the composition and location of primary spinal cord tumors. Despite being a rare diagnosis, meticulous attention by physicians to related presentations might lead to a decreased delay in diagnosis, thereby reducing insidious outcomes brought about by the progression of the tumors.

## Authors’ Contribution

Erfan Tasdighi was responsible for data acquisition. Abdonaser Farzan was the surgeon and designed the study. Pooria Ahmadi interpreted the data and discussed the results. Elham Pourbakhtyaran drafted the manuscript and granted the final approval of the version to be published.

All authors agreed to be accountable for all aspects of the work to ensure that questions related to the accuracy or integrity of any part of the work are appropriately investigated and resolved.

## Conflicts of Interest

The authors disclose no conflict of interest.
